# The roles and challenges of the primary health care systems in epidemic management: a scoping review

**DOI:** 10.1017/S1463423623000452

**Published:** 2023-09-14

**Authors:** Elham Shami, Kamal Gholipour, Deniz Naghibi, Saber Azami-Aghdash

**Affiliations:** 1 Iranian Center of Excellence in Health Management, Department of Health Services Management, School of Management and Medical Informatics, Tabriz University of Medical Sciences, Tabriz, Iran; 2 Social Determinants of Health Research Center, Department of Health Service Management, School of Management and Medical Informatics, Tabriz University of Medical Sciences, Tabriz, Iran; 3 Student Research Committee, Tabriz University of Medical Sciences, Tabriz, Iran; 4 Tabriz Health Services Management Research Center, School of Management and Medical Informatics, Tabriz University of Medical Sciences, Tabriz, Iran

**Keywords:** challenge, epidemics, infectious disease, management, primary health care, role

## Abstract

**Background and aim:**

During the early stage of pandemics, primary health care (PHC) is the first point of contact with the health system for people. This study aimed to find the leading roles and challenges of the PHC system in dealing with the outbreak of infectious diseases

**Methods:**

The current scoping review was conducted in 2022 using the Arkesy and O’Malley framework. A bibliographic search was conducted in PubMed, Web of Science, and Scopus databases. Following a Google Scholar search, a manual search in some journals, reference checks for articles, and a review of organizational reports, websites, and other sources of information were also conducted. Data were analyzed using the content-analysis method.

**Findings:**

Finally, 65 documents (42 articles and 23 reports, books, and news) were included in the study. Initially, 626 codes were extracted, and 132 final codes were categorized into eight main themes and 44 sub-themes. The main themes for the roles of PHC included: service provision, education and knowledge, surveillance, access, coordination and communication, management and leadership, infrastructure change and rapid preparation, and patient and community management. Regarding the challenges faced by PHC in the epidemic of infectious diseases, 24 key challenges were identified and categorized into four major areas.

**Conclusions:**

Based on the results of the present study, there is a need for further studies to formulate and theorize the specific roles of PHC in managing infectious disease epidemics. The results of this study can be utilized by researchers and officials to inform their efforts in addressing this purpose.

## Introduction

Primary health care (PHC) is the pillar of an available and cost-effective health system. Moreover, PHC is a central component of healthcare and the first point of contact with people and society (Starfield et al., 2005). The role of PHC in the healthcare system is more essential than ever during epidemics and pandemics as the first line of defense (Starfield B). PHC assumes pivotal roles in such scenarios, serving as the gatekeeper of the health system and spearheading preventive measures to combat the spread of infectious diseases (Patey et al., 2020). Over 95% of all healthcare activities are handled by PHC (Dunlop et al., 2020). When community-wide epidemics of COVID-like illnesses, caused by a novel virus with an unknown level of virulence, emerge, it becomes imperative to enhance the capacity of PHC services. During this time, PHC potentially provides accessible health services, continuity of care, an available multi-skilled workforce, and a triage mechanism to refer patients to tertiary care (Ashley et al., 2021).

Infectious disease outbreaks are increasingly prevalent, with COVID-19 being the most significant and impactful epidemic of this century, marking the fifth major infectious disease outbreak (De Lusignan et al., 2017). PHC providers, including those in primary care and public health roles, have critical responsibilities in managing healthcare emergencies. Primary care requires robust epidemiological foundation and the utilization of essential monitoring and surveillance systems to detect disease outbreaks promptly (De Lusignan et al., 2017).

While PHC bears the primary responsibility for addressing infectious disease epidemics, the specific roles it should assume in epidemic management remain unclear. This lack of clarity often leads to delays and inadequate responses in dealing with epidemics, as well as instances of overlapping roles among different healthcare entities. Amidst recent global epidemics, only a handful of countries have demonstrated the ability to provide prompt and accurate responses in effectively addressing the COVID-19 pandemic. It seems logical that having a clear picture and sufficient information about the roles of PHC can play a pivotal role in the effective management of infectious diseases epidemic crises. Therefore, the aim of the present study is to clarify and describe the central roles of PHC in ensuring a swift and effective response to epidemics, as well as challenges faced by the PHC system in effectively managing outbreaks of infectious diseases.

## Method

### Search strategy

This study was conducted based on the framework developed by Arksey and O’Malley (Arksey and O’Malley, 2005, Levac et al., 2010). This framework consists of six stages: (a) defining the research question, (b) identifying relevant studies or search strategy, (c) selecting studies, (d) charting the data and assessing the quality of studies included, (e) collating, summarizing, and reporting the data, and (f) consultation (Arksey and O’Malley, 2005).

The inclusion criteria consisted of examining the roles or challenges of PHC in pandemics and epidemics of various viral and infectious diseases, such as MERS, SARS, COVID-19, and H1N1, describing the role of the family physician in epidemics, and studying different countries’ experiences with the performance of PHC in a pandemic and epidemic of infectious diseases and challenges faced by PHC. The inclusion criteria for this study were limited to research conducted and published in the English language.

Studies discussing the epidemiology of viral diseases and the studies conducted in hospitals and other private centers were excluded.

The current study’s main question is ‘What are PHC’s roles and challenges in managing an infectious disease epidemic?’ The sub-questions include the following:

- What are the challenges of infectious disease epidemic management faced by PHC systems?
- What are the roles of the PHC system in dealing with an infectious disease epidemic?

### Identification of relevant studies

The literature search was performed across Scopus, PubMed, and Web of Science databases. Some reports were retrieved from the Google Scholar search engine and health organization websites. An initial search in several databases was conducted to identify articles on the topic. The keywords in the titles and abstracts of relevant articles were used to develop a complete and refined search strategy for the target databases. PubMed, Web of Science, and Scopus were searched based on the established search strategy on 24 May 2022. The search strings consisted of a combination of keywords including PHC, primary care, primary health, primary healthcare, public health, role, function, upgrade performance, infectious disease, communicable diseases, contagious diseases, pandemics, diseases outbreaks, community, rural, family physician, family medicine, basic health care, actions, initiative, and activity (Additional file1).

To conduct gray literature search, the databases of the European Association for Gray Literature Exploitation, Health Care Management Information Consortium, and System for Information on Grey Literature in Europe were searched. Following the exclusion of articles that had weak relevance to the study objectives, the reference lists of the selected articles were also scrutinized for additional relevant sources to ensure a higher level of confidence in the identification and review of resources. A citation check was also done for the selected articles through Google Scholar. More searches were done through Google search engine. Other sources of information were reported in additional file 2.

Other sources of information we searched were as follows:

- Reviewing websites of the World Health Organization (WHO)
- Reviewing websites of international organizations such as the Primary Health Care Performance Initiative
- Finding all published reports on the role of PHC in epidemic management.

### Study selection

Two research team members performed all article screening and selection stages independently. In case of disagreement, members reached to an agreement through discussion. If necessary, disputes were referred to a third party with more information and experience.

To begin with, the titles of all articles were reviewed, leading to the exclusion of papers inconsistent with the study objectives. The articles’ abstracts and full texts were studied in the following steps. Accordingly, studies that met exclusion criteria and those with a weak association with the study aims were identified and excluded.

**Figure 1.**
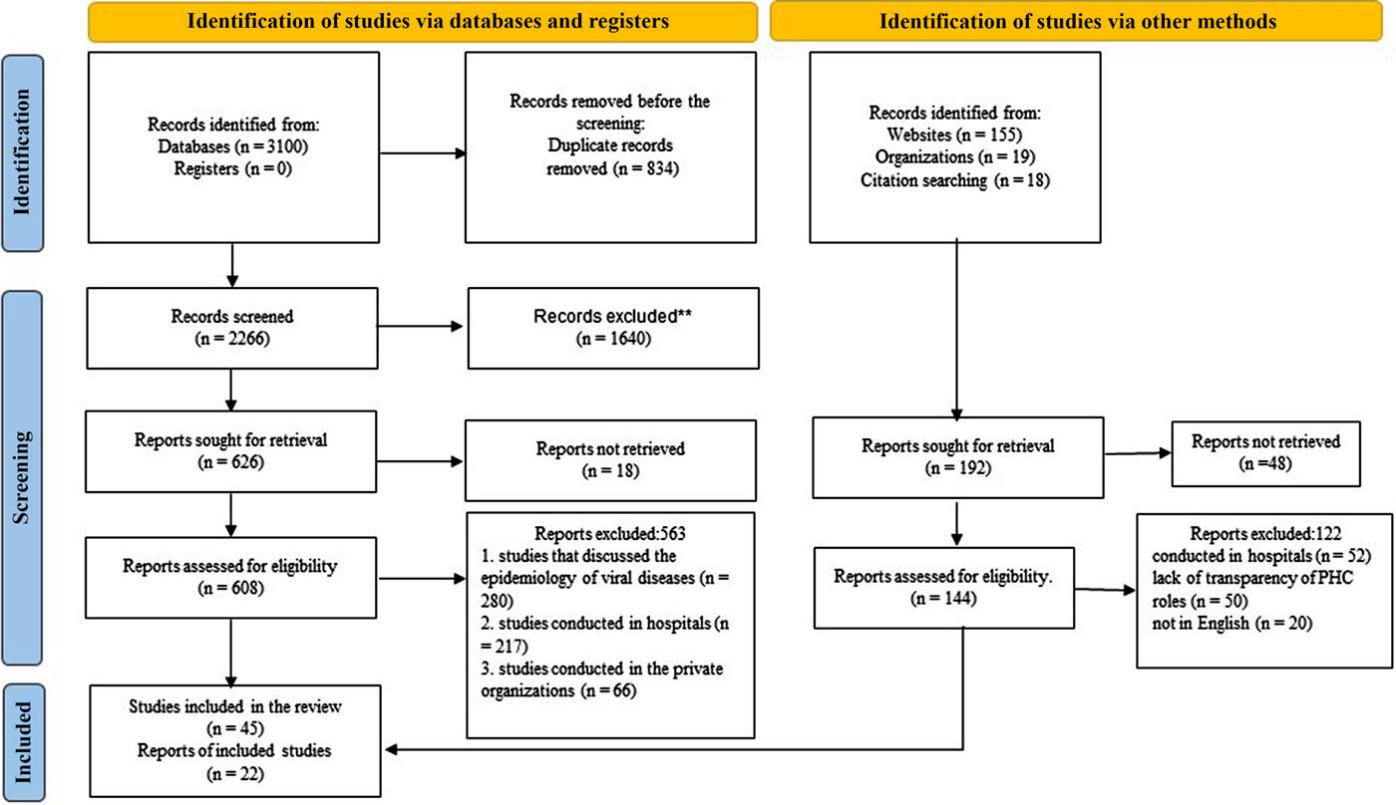
PRISMA-based flow diagram

Given that the structure of PHC delivery varies from country to country, some measures were taken to determine whether the defined roles are parts of PHC. Two researchers examined the information provided in each study and whether it was related to PHC. Endnote X8 reference management software was used to organize references, study titles, and abstracts and identify duplicates. The PRISMA 2020 flowchart was used to report the study selection and screening process (Figure 1).

### Data charting

The data charting form was designed in Microsoft Excel 2010 and piloted by two authors using the data from five articles. All results were entered into the data charting form during the data extraction. This form encompassed study characteristics (i.e., author, country, purpose of the study, study design, the role of PHC in pandemics, preliminary results, and other information). Finally, two researchers independently extracted the information from the included articles, blinded to each other’s findings. Then the ambiguities were resolved in discussion with the research team.

### Data extraction and synthesis

**Figure 2.**
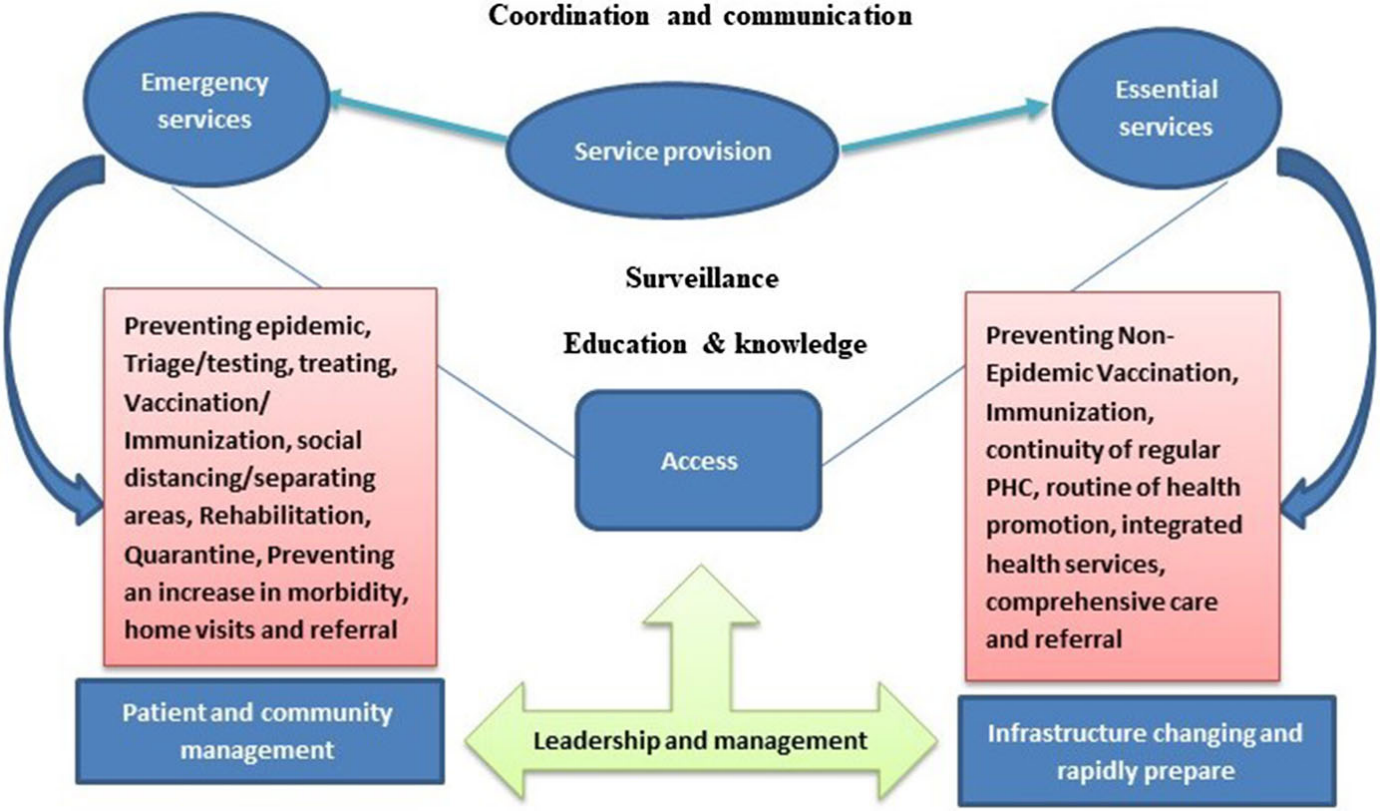
The primary roles of PHC in the management of epidemics and pandemics of infectious disease

Content-analysis method was applied to analyze data by two researchers independently. The data analysis process was conducted in five steps: (1) Familiarity with the text of papers (immersion in the articles), (2) Identifying the categories, (3) Placement of articles in specified categories, (4) Reviewing the article categorization based on their results, and (5) Ensuring the results’ reliability through consensus. The main roles extracted from the studies were presented under general headings. Then each one was analyzed, and the relationships between them were determined (Figure 2).

## Results

### Screening results

A total of 3100 studies was obtained from the database search; 834 records were excluded due to duplication. Based on the title and abstract screening results, 1640 records were excluded. Eighteen records were not retrieved, and 563 studies were excluded in the full-text screening stage. Finally, 45 articles were included in the study. Researchers also searched for the reports, and 22 cases were reviewed. Finally, 67 studies and reports met the inclusion criteria (Figure 1).

### General characteristics of reviewed studies

The included studies’ publication dates ranged from 2007 to 2022. Most studies were published in 2020 (*n* = 28) and 2021 (*n* = 21). Most studies were conducted in Australia, the United Kingdom, Brazil, China, and India. Content extracted from reports and articles was categorized into eight categories: service provision, education and knowledge, surveillance, access, coordination and communication, management and leadership, infrastructure change and rapid preparation, and patient and community management (Figure 2).

The themes obtained from the studies and reports were reviewed several times. As a result, the repeated items were merged, and the remaining items were placed in specific categories. Table 1 shows the roles of PHC in dealing with the epidemic of infectious diseases. Each topic is divided into subtopics in this table, with examples.

**Table 1.** The role of PHC in dealing with an infectious disease epidemic (themes and sub-themes)

Theme	Sub-theme
Access	Geographical access {McQuilkin, 2017 #92}{Sarti, 2020 #174}{Haldane, 2020 #128}{Daumas, 2020 #96}
	Availability {Kyle, 2021 #97}{Agnello, 2016 #153}
Disease surveillance	Monitoring {Sarti, 2020 #174}{Gössling, 2020 #100}{Dunlop, 2020 #137}
	Case finding {Kyle, 2021 #97}{Prado, 2020 #104}{Siedner, 2020 #105}
	Contact tracing {Pan et al., 2020 #107;Medina, 2020 #108;Xu, 2020 #109;Pan et al., 2020 #138;Medina, 2020 #150;Xu, 2020 #116}
	Monitoring for early signs of an outbreak {Xu, 2020 #116}
	Surveillance in community and household settings {Dunlop, 2020 #40}{Medina, 2020 #150}{Wong, 2021 #30}{Phillips, 2007 #113}
	Surveillance at checkpoints {Siedner, 2020 #117}{Xu, 2020 #115}{Ahmed, 2020 #118}{Zhu, 2021 #123}
Coordination and communication	Facilitate within PHC coordination and communication {Sarti, 2020 #101}{Wong, 2021 #120}{Zhu, 2021 #123}{Rawaf, 2020 #130}
	Facilitate within the health sector {Sarti, 2020 #122}{Wong, 2021 #120}{Rawaf, 2020 #130}
	Facilitate coordination and communication between other sectors {Sarti, 2020 #102}{Wong, 2021 #120}{Zhu, 2021 #123}{Rawaf, 2020 #130}
Infrastructure adaptation	Use modern digital technologies {Rawaf, 2020 #130}
	Establishment of special epidemic management centers {Daumas, 2020 #126}
	Modify centers’ structure {Sarti, 2020 #175}
Service provision	Emergency service delivery {Haldane, 2020 #128}{Harnden, 2021 #127}{Park, 2020 #145}
	Routine service delivery {Agnello, 2016 #98}{Dunlop, 2020 #103}{Rawaf, 2020 #121}
	Disease control {Haldane, 2020 #148}{Pan et al., 2020 #138}{Mazowita, 2006 #133}
Education & knowledge	Epidemic specific training {Dunlop, 2020 #141}{Alakeely, 2021 #136}
	General training {Pan et al., 2020 #107}{Rawaf, 2020 #29}{Mazowita, 2006 #140}
Management and leadership	Prioritization of services/resources {Mazowita, 2006 #140}
	Provide proper preparedness plans for pandemics {Dunlop, 2020 #37}{Wong, 2021 #30}{Lee, 2010 #142}
	Improve quality {Kearon, 2020 #144}
	Integrated planning/change management {Park, 2020 #145;Park, 2020 #129}
	Performance assessment {Zhu, 2021 #146}
	Human resources management {Alakeely, 2021 #136}
	Evidence-based decision making {Haldane, 2020 #128}
	Referral system {Prado, 2020 #104}
Patient and community management	Comprehensive patient support system {Medina, 2020 #150;Daumas, 2020 #151;Daumas, 2020 #151}{Medina, 2020 #114}
	Proactive engagement with communities {Windak, 2022 #152;Agnello, 2016 #153;Agnello, 2016 #153}{Windak, 2022 #152}
	Social mobilization {Boyce, 2019 #154}

### Roles of PHC in dealing with an infectious disease epidemic

In this figure, the essential roles of PHC are illustrated. PHC plays a vital role in improving people’s health by providing services and primary care indirectly. On the other hand, it plays the most crucial direct role in identifying and diagnosing patients. Due to the high prevalence of the disease and the speed of the virus spread, epidemics and pandemics require a robust system that can detect the disease promptly. Moreover, to slow down the disease spread, the health system needs cooperation and coordination within the entire country, such as the police force.

Additionally, educating people about the epidemic, transmission, and prevention can be very effective in reducing the speed of the disease’s spread. People should trust PHC and follow the updates and announcements only through this channel. The current structure of PHC is inadequate in effectively responding to epidemics of infectious diseases. Consequently, a restructuring of this system is imperative to accommodate the physical space required for patient care, patient isolation, and routine care for healthy individuals. Furthermore, there is a pressing need to ensure that the PHC system possesses the necessary human resources to cope with the escalating demand for patient care during epidemics.

### Coordination and communication

One of the essential components of PHC is coordination. This principle seems more critical in the management of infectious disease pandemics. The existing literature divided coordination into two types: intra-partial and extra-partial. Internal coordination refers to the coordination within the health system, such as the coordination of PHC with hospitals. In coordination with the local authorities, additional sites (e.g., convalescent homes, hotels, schools, community centers, and gymnasiums) could be converted into patient care units (Rust et al., 2009). On the other hand, external coordination refers to the coordination between different organizations and the health system, such as municipal coordination with health (Desborough et al., 2020). Since the epidemic of infectious diseases leads to an increased demand for PHCs, more intra-sectoral coordination between the hospitals and PHCs is necessary (Yan et al., 2022).

### Service provision

Providing services at the first level of the health system is the responsibility of PHC system. Our study categorized the provision of PHC services into two groups: essential services and emergency services. Essential services include prevention, vaccination/ immunization, continuity of regular PHC, routine health promotion, integrated health services, comprehensive care, and referral system. These services could be in the form of maternal and infant care, care for the elderly, care for non-communicable diseases, care for infectious diseases such as tuberculosis, and environmental health. Providing emergency services includes preventing epidemics, triage/testing, treating, vaccination/ immunization, social distancing/separating areas, rehabilitation, quarantine, preventing an increase in morbidity, home visits, and referral. PHC should maintain essential services and effectively assist in controlling the pandemic.

In pandemics, primary care potentially provides accessible health services, continuity of care, an available multi-skilled workforce, and a triage mechanism (Dawad and Jobson, 2011). However, the isolation in epidemics should be emphasized for patients with viral diseases (Siddiqui et al., 2019) (Chia et al., 2021).

### Education & knowledge

One of the responsibilities of PHC is to educate people. In this regard, improving people’s knowledge about infectious diseases and ways of virus transmission from person to person is essential. Existing studies have categorized education into subcategories, distinguishing between educational concepts specifically tailored for the epidemic period and education applicable to non-epidemic situations, including raising awareness.

PHC can play an essential role in educating people living in the deprived parts of the metropolitan areas, where there is a significant lack of awareness regarding the unprecedented situation. PHC staff could educate individuals about the importance of social distancing, covering faces, and sanitizing hands, to name a few. They can also advise people when to seek medical attention and what symptoms to look for (Mukhamedyarova et al., 2021). The public is currently overwhelmed with epidemiological data disseminated by the media, often with little sense of their quality, caveats, and risk adjustment issues (Rawaf et al., 2020).

### Surveillance

Infectious disease surveillance helps a lot in managing infectious disease epidemics. Surveillance consists of monitoring, control-case surveillance, contact tracing, monitoring for early signs of an outbreak, surveillance in community and household settings, surveillance at checkpoints, and observation.

Primary care physicians play a pivotal role within surveillance systems as they bear the responsibility of promptly reporting significant communicable diseases to public health authorities. For example, as a patient’s first point of contact, primary care tends to identify a spike in seasonal influenza earlier than in emergency departments and can serve as a reliable indicator of underlying trends in community transmission (Rust et al., 2009; Sloane et al., 2006).

### Access

Access to good health is one of the PHC systems’ roles in infectious disease pandemics. In this regard, access consists of two sub-themes: geographical access and availability. Primary care also plays a pivotal role in health systems to enable universal access to care. For instance, physicians’ ability in primary care has directly affected the overall management of both infectious and chronic respiratory diseases in China (Pan et al., 2020).

The utilization of new technologies in diagnosing and caring for patients during the infectious diseases pandemic can significantly enhance people’s access to healthcare services. Mobile pharmacy programs use an online platform to streamline the process of getting medication refills to reduce contact time and the risk of cross-infection (Yan et al., 2022). According to WHO, the number of people who died from reduced access to usual care probably surpassed the number of deaths directly caused by the virus itself (Dunlop et al., 2020).

### Management and leadership

PHC has the responsibility of managing a defined population and specific area. The items included in this theme are as follows: prioritization, establishing trust, organization, preparedness for a pandemic, quality improvement, responses, integrated planning/change management, and control for PHC institutions.

Evidence-based decision-making is an essential element of management. The use of evidence-based information and protocols influences the decisions of health policymakers in epidemic management. Evidence-based information includes protocols, instructions, policies, epidemiological reports, and clinical evidence (Iezadi et al., 2021).

Human resource management is the most critical component of health management. The workforce is the most crucial resource of the health system; therefore, taking care of them is one of the main functions of health systems. Caring for human resources is also critical in the infectious disease epidemic. This theme is classified into subcategories of protecting the workforce, comprehensive response measures, and ensuring staff safety. Health systems should be prepared to ensure additional equipment and staff to meet demands and prevent burnout (Haldane et al., 2020).

### Infrastructure change and rapid preparation

Epidemics demand the establishment of a robust infrastructure in various domains within the realm of PHC. Adequate planning and preparation are essential to develop and reinforce this infrastructure. The subcategories include new technologies, the establishment of special epidemic centers, and restructuring.

For example, it is necessary to set up a dedicated space for screening/triage for all visitors and patients in all health facilities and ensure that the space has a dedicated waiting area. After that, it is essential to take measures in the waiting area: hand hygiene, physical distancing, mask-wearing, and visible signage/instructions for patient flow.

Making a dedicated isolation room available during the health facility’s opening hours is essential. It can be either an area of the existing infrastructure or a temporary structure allocated to this purpose and must be located away from waiting areas and consultation rooms (Rust et al., 2009).

### Patient and community management

This concept includes sub-themes of social support, supporting patients physically, psychological support, support communities, isolating suspected cases, proactive engagement with communities, social mobilization, acting as community-level educators, addressing fear and concern among the staff and in the community, keeping communities safe and healthy, increasing trust in services, and crossing management.

### PHC challenges in epidemics

The current study obtained some of the problems and challenges faced by PHC in dealing with the sudden epidemic and pandemic of infectious diseases. The number of challenges mentioned in the included articles was 24 items, among which some were mentioned in most of the studies. One of the most critical challenges in dealing with different epidemics was the shortage of personal protective equipment (PPE) at the beginning of the outbreak (Wong et al., 2007).

Another challenge was the lack of public understanding and trust in healthcare organizations, including health centers and hospitals. Additionally, staff and people’s fear of visiting healthcare facilities due to potential exposure to illnesses was also a significant concern. The centers faced fear and dread, making people neglect essential services such as maternal and neonatal care services, chronic and non-communicable disease care, or care for the elderly. Furthermore, this cycle causes an increase in non-communicable diseases and the emergence of another crisis after the epidemic (Wong et al., 2021).

The most fundamental challenge was the lack of coordination between PHC and hospitals. On the other hand, weak coordination and collaboration between various organizations and PHC further complicated the management of the epidemic (Haldane et al., 2020).

The absence of adequate planning and preparation for infectious disease epidemics resulted in countries experiencing a sudden surge in the number of patients and the rate of disease outbreaks, which led to a rise in the mortality rate. Furthermore, disease prevention and epidemic preparedness were not given sufficient attention. Numerous studies have highlighted the absence of comprehensive plans or guidelines for the care and treatment of infected patients. This lack of standardized protocols led healthcare providers to make decisions based on personal preferences rather than evidence-based practices (Desborough et al., 2021a).

At the health center level, challenges included the burden of paperwork, managing information, and handling statistics, which significantly increased the workload; challenges to make patient care a top priority; the shortage of human resources in the PHC system; and insufficient training to cope with the existing situation. The epidemics caused confusion in the workforce, while simultaneously, the surge in demand for healthcare services in the medical facilities led to an overwhelming workload and burnout among employees (Xu et al., 2020) (Table 2).

**Table 2.** Challenges and upgradable points faced by PHC during epidemics

Domains	Challenges
Management	Changes needed in PHC financing and organization
	Lack of guidance to support the supply chain
	Lack of clear structures/guidelines for case definition and treatment
	Need to set up a temporary quarantine space
	Lack of communication or miscommunication between public health agencies and primary health care
	Drawbacks in coordination between primary care and hospitals
	No referral system
	Paperwork
Material	Lack of skills in using new technology
	Heavy workload
	Lack of personal protective equipment (PPE) or space
	Shortage of the staff
	Lack of telehealth knowledge
	Lack of guidelines specific to COVID-19
Patient behavior	Lack of trust and co-understanding in people
	Fear of visiting health centers (face-to-face visits)
	Restrictions on people’s movements (causing mental disorders)
Delivery of health services	Delay in routine care
	Lack of coordination between different sectors
	Lack of a plan to guide referral
	Lack of consensus on the best model for intervention/immunization
	Fear and lack of knowledge related to the pandemic among patients and staff

## Discussion

The summary of the study results shows that epidemics and pandemics necessitate swift responses from both PHC systems and communities to adapt to a novel and uncertain clinical and epidemiological environment, with a potentially high risk of morbidity and mortality.

In this review, we extracted eight key themes for PHC roles, including service provision, education and knowledge, surveillance, access, coordination and communication, management and leadership, infrastructure change and rapid preparation, and patient and community management for PHC during infectious disease outbreaks. Accordingly, infrastructure change, rapid preparation, coordination, and communication are the most necessary measures in epidemics. It is essential to plan and prepare before the epidemic outbreak. In this regard, it is essential to identify the prominent roles of PHC during epidemics for proper planning. With regard to the leading roles of PHC, we can point out the foundation of changes in system structures, training, and inter-departmental cooperation suiting new structures.

WHO has categorized PHC roles in the COVID-19 pandemic into five categories. The WHO report mentions more political issues in dealing with the epidemic. Meanwhile, our study pointed to the primary roles of PHC at the executive level (World Health Organization. Regional Office for the Western Pacific, 2020). Another study categorized PHC roles into eight groups pointing out the importance of education; communication; and inter-departmental cooperation, identification, and consideration of patient surveillance, triage, and treatment of illness in the community during a pandemic, preventing the spread amongst vulnerable populations, home and community care of higher acuity non-pandemic illness, vaccination, post-pandemic recovery, and pandemic planning (Hartzler et al., 2018).

Defining the role of PHC in a pandemic is a responsibility that countries must undertake, as emphasized by the WHO. The aim of our study was to ascertain the role of PHC in managing epidemics of infectious diseases. This research builds upon previous studies that have highlighted the necessity of defining the role of PHC in responding to infectious disease outbreaks, including those related to SARS, H1N1, and EVD. Lack of role clarity was a source of distress for family physicians during the SARS outbreaks in Ontario (Desborough et al., 2021b, Austin et al., 2007). A study in Australia also mentioned the need to clarify the role of PHC. Following H1N1, the Canadian primary care system described ambiguity related to a lack of clarity in pandemic influenza planning and logistical issues. In contrast, staff working in centers with more detailed information reported less stress and fewer unforeseen problems and delays (Masotti et al., 2013). Australian PHCs perceived conflict between the PHC role and usual clinical care responsibilities, primarily driven by a lack of capacity to perform both (Li et al., 2021, Bocquet et al., 2010).

Based on our study results, determining knowledge and guidelines is necessary for overcoming epidemics, which is confirmed by other studies. During the SARS outbreak, family physicians in Ontario reported not having the appropriate knowledge and skills to protect themselves, their patients, staff, and their families (Desborough et al., 2021b). On the other hand, the cancelation of medical education events increased their sense of professional isolation. Surveys investigating SARS and H1N1 influenza found that most PHCs had either no or insufficient training in infectious disease control, lacked confidence, and required education to inform disease management (James et al., 2006).

In this study, one of the most critical roles of PHC was considered to be service provision. In an epidemic, the health system faces an extra duty because, in addition to providing routine services, it also has to provide services to support the population. This situation puts a heavy burden on PHC and may reduce the quality or quantity of services. The decline in non-COVID-19-related care raised concerns about the quality of essential care for patients with chronic health problems, such as proactive care for diabetes (Sarma et al., 2020). According to a previous study, the COVID-19 pandemic has taken a heavy toll on essential health services and unbalanced its already reduced capacity to deliver essential health services since April 2020 (Romero-Martínez et al., 2013). Previous studies have shown that sick children visits, childhood vaccinations, contraceptive users, and other health services had a downward trend in 2019, and the COVID-19 pandemic accelerated these negative trends. The pandemic also heavily affected other services, such as cervical and breast cancer screening, in-person diabetes and hypertension visits, and elective surgeries (Ejecutivo et al.,; Doubova et al., 2021). Another previous study showed that many PHC services promptly implemented mechanisms to balance the availability of services to keep patients and staff safe and retain essential and urgent services unrelated to COVID-19 (Ashley et al., 2021).

As per our study findings, the key recommendation is for primary care to demonstrate the ability to swiftly adapt and innovate by promptly redirecting patient flow, as it stands as a primary area of focus during infectious disease epidemics (Ashley et al., 2021). Despite the challenges, it is noteworthy that primary care has achieved significant success in this regard, often with limited external support (Greenhalgh et al., 2020). Accordingly, Singapore’s exemplary approach to managing infectious disease outbreaks lies in its community health centers, which possess fully equipped consulting rooms that can be isolated from the rest of the practice. Additionally, these centers have separate entrances accessible by ambulance and stretcher, ensuring efficient patient transport and containment of potential infections (Nanjunda, 2011, Rawaf et al., 2020).

Another lesson learned from this review is related to the collection and dissemination of finely tuned information that can fit the goals. Based on a previous study, information users (the public, patients, clinicians, and policymakers) all have information needs that cannot be served by the same measures (Rawaf et al., 2020). The public is currently overwhelmed with epidemiological data disseminated by the media, often with little sense of their quality, caveats, and risk adjustment issues. Efforts must be directed toward addressing the public’s information needs in a comprehensible manner, considering people’s fears about the spread of the virus and countering the dissemination of fake news or misleading data. These challenges are likely to accompany every new pandemic and can have a significant impact on public perceptions and behaviors (Wind et al., 2020, Rawaf et al., 2020).

In this study, we also categorized challenges faced by PHC during emerging epidemics, the most important of which were lack of planning, lack of infrastructure, and weakness in inter-sectoral cooperation. We classified the challenges into four main categories: management and infrastructure, material, patient behavior, and delivery of health services. This study showed that PHC faces challenges in dealing with epidemics that lead to poor performance and reduced responsiveness. The most common challenge that the literature mentioned was the delay in routine care (Gupta and Denton, 2008). The provision of essential healthcare services during the COVID-19 pandemic is a pivotal challenge that could impose a double burden on the already exhausted PHC (Garg et al., 2020). UNICEF estimates that nearly 117 million children from 37 countries are at risk of missing the life-saving measles vaccine (Figueroa, 2020). A study reported that the epidemic in West Africa during the 2014–2016 Ebola outbreak resulted in nearly 10 000 preventable deaths due to malaria, HIV/AIDS, and tuberculosis (Parpia et al., 2016). Another critical challenge is the lack of necessary infrastructure for respiratory diseases such as COVID-19. For example, there is a need for ventilation and space to maintain physical distancing. Separate spaces for affected patients are needed for screening, testing, and surveillance (Maves et al., 2020, Iyengar et al., 2020). In this study, the lack of cooperation and coordination was identified as one of the most complex and significant challenges in dealing with epidemics. Previous studies highlighted the need to improve collaboration between PHC and hospitals (Wong et al., 2012), between different levels of government (Iyengar et al., 2015, Wong et al., 2007), and between public and private sectors (Wong et al., 2007, Wong et al., 2004). Additionally, protecting employees has been addressed as one of the challenges in previous studies. Keeping the healthcare workforce from harm is critical during a pandemic. Despite early success in suppressing COVID-19 transmission, approximately 15% of cases in Victoria, Australia, in August 2020 were of healthcare workers (Scott et al., 2020). Lack of access to appropriate PPE, lack of PHC-relevant training and guidelines impacting safe work practices, and lack of transparent information from reliable sources are other challenges faced by the healthcare workforce (Pappa et al., 2020). People’s fear and refusal to go to health centers were other challenges. Other studies showed that diseases have expanded due to the fear of facing the illness, and the care of patients in the post-epidemic period is the responsibility of family physicians (Galea et al., 2020). In addition, previous studies show that service providers are afraid to face patients, be exposed to infectious agents, and transmit the infection to others (Grayson and Johnson, 2009). One of the less frequently mentioned (Abdoul-Azize and El Gamil, 2021) (Subba et al., 2021), but highly significant, challenges during epidemics is the issue of financing (Ramanathan et al., 2020). A study has highlighted the significance of preparing financing for PHC during times of crisis from two critical perspectives. Firstly, adequate financing is essential to effectively respond to epidemics, ensuring that healthcare systems have the necessary resources. Secondly, robust financing is vital for maintaining the resilience of the health system throughout the epidemic (Barbisch and Koenig, 2006). In another study, the importance of financing during urgent and critical periods in the health sector has been underscored. Adequate funding is crucial not only to cover the essential costs for health workers to motivate them but also to maintain their dedication and commitment to caring for patients during crises (Ramanathan et al., 2020).

The present study has comprehensively summarized and analyzed the available information and evidence on the roles and challenges of PHC in the management of infectious disease epidemics thus providing extensive and practical information for readers and decision makers. Nonetheless, this study encountered several limitations, one of the most significant being the restricted access to information and experiences from different countries. The possible cause of this limitation could be the lack of information dissemination or its release in local languages rather than English, making it inaccessible to the researchers.

### Conclusion

In this study, the roles and challenges of PHC in managing infectious disease epidemics were identified, categorized, and analyzed by reviewing experiences from different countries and existing literature. The findings of this research can serve as valuable insights for decision-makers in enhancing management strategies and policymaking during epidemics. It is worth noting that the number of studies specifically focusing on developing these roles is limited. As a result, future research is recommended to prioritize the development of specific models or theories in this field.
